# Cardiovascular effects of intravenous morphine in anesthetized horse

**DOI:** 10.3389/fvets.2022.1007345

**Published:** 2022-09-26

**Authors:** Emma Hoeberg, Henning Andreas Haga, Andreas Lervik

**Affiliations:** Department of Companion Animal Clinical Sciences, Norwegian University of Life Sciences, Ås, Norway

**Keywords:** anesthesia, cardiovascular effect, horse, hypotension, morphine

## Abstract

**Objectives:**

To investigate whether morphine causes a change in mean arterial blood pressure (MAP) heart rate (HR) and oxygen extraction (OE) rate in healthy horses anesthetized with isoflurane and a dexmedetomidine infusion.

**Material and methods:**

The study design was prospective clinical, randomized, blinded two groups including 33 horses. All horses were sedated with romifidine IV, and anesthesia was induced with midazolam IV and ketamine IV and maintained with isoflurane in oxygen and medical air and a dexmedetomidine infusion. As a baseline venous and arterial blood, HR and MAP were sampled. Thereafter either morphine 0.1 mg kg^−1^ IV or an equivalent volume of NaCl 0.9% IV was administered. HR and MAP were then further sampled for 5 min before venous and arterial blood was again sampled. OE was calculated based upon arterial and venous blood gas analysis. To evaluate the change in minimum MAP, mean HR, and OE, the differences between baseline and observation period values were further termed delta MAP, delta HR, and delta OE. Individual delta MAPs were normalized to the minimum baseline value and are reported as a percentage. Alpha was set to 0.05. Confidence intervals 95% (CI) were calculated for delta MAP, delta HR, and delta OE within groups, and for the difference between groups.

**Results:**

The 95% CIs for delta MAP (%), delta HR (min^−1^), and delta OE (mL/dL) in the morphine group were −20.5 to −9.0, 0.6 to 3.1, and −0.1 to 0.6 and in the placebo group were −17.4 to −10.1, 0.2 to 2.0, and −0.2 to 0.3, respectively. The 95% CI for the differences in delta MAP (%), delta HR (min^−1^), and delta OE (mL/dL) were −5.5 to 7.6, −2.3 to 0.7, and −0.7 to 0.2, respectively. The minimum MAP of one horse in the morphine group decreased around 50% between baseline and observation period with almost unchanged OE and HR.

**Conclusion and clinical relevance:**

The effects of morphine 0.1 mg kg^−1^ IV on HR, MAP, and OE in healthy horses anesthetized with isoflurane and a CRI of dexmedetomidine are minimal.

## Introduction

Morphine is a μ agonist with some κ agonist effect (1). It is used as an analgesic in different species (1) and is often a part of balanced anesthesia in horses (2). Opioids may produce cardiovascular changes in mammals, and in dogs, bradycardia (1) and increased plasma histamine concentration are potential causes of hypotension (1, 3). In cats, increased histamine release and decreased blood pressure have been described (4, 5). The cardiovascular effect of intravenous morphine in horses has been investigated to some extent (2, 6–11). Three studies have investigated the cardiovascular response to morphine in conscious horses, reporting increases in heart rate (HR) and invasive blood pressure (IBP) (6, 7, 10). Most of the studies examining the cardiovascular response to morphine during general anesthesia in horses did not detect a significant difference in IBP and HR between horses receiving morphine before or during anesthesia (8), during anesthesia (2, 9) or when different doses were administered during anesthesia (2). However, one study found a significant decrease in mean arterial blood pressure (MAP) and increase in oxygen extraction (OE) ratio but no change in HR after administration of morphine 0.2 mg kg^−1^ (11). One study in horses investigated immediate cardiovascular changes by recording cardiovascular variables only once 2 min after injection of morphine (8). Also, studies in other species indicate an immediate cardiovascular change (1, 3–5). Therefore, it is of interest to continually measure the immediate effect an intravenous injection of morphine has on the cardiovascular system in horses.

A highly important part of equine anesthesia is to secure adequate perfusion of the large muscles and thereby tissue oxygenation (12, 13), as an impairment might have a negative impact on survival rate of horses during general anesthesia (14). Cardiac output (Qt) is the product of stroke volume and HR but is rarely measured in equine patients during anesthesia. Measurement of IBP is often taken as an indicator of tissue perfusion, but this parameter does not always reflect blood flow and oxygen delivery (12, 13). Insufficient oxygen tissue supply can result from globally decreased delivery of oxygen (DO_2_) or, as is the case in anesthetized horses, decreased muscle perfusion pressure. If Qt decreases and oxygen consumption (VO_2_) is unchanged, OE will increase as long as DO_2_ meets the VO_2_ requirements (15, 16). Therefore, in an anesthetized animal where VO_2_ likely is stable and DO_2_ is adequate, OE may be used as a surrogate measure for reflection of changes in Qt. To evaluate the effect of intravenous morphine on cardiovascular function in anesthetized horses, we decided to measure OE in combination with IBP and HR.

The primary objective of this study was to investigate if morphine causes a change in MAP, and secondary was to investigate the effect of morphine upon HR and OE in healthy horses anesthetized with isoflurane and a dexmedetomidine infusion.

## Materials and methods

The study was approved by the National Animal Research Authority, FOTS id 14/04723-66. A prospective clinical, randomized, blinded two groups study was performed. An a priori power analysis was performed using a standard deviation of 5.8 mmHg based upon a pilot study including five horses, a beta of 0.8, an alpha of 0.05, and a minimum effect size of 6 mm Hg resulting in a total sample size of 32 horses. Horses were randomized into two groups based upon permuted block randomization in blocks of four horses prior to study start.

Adult (≥ 1 year) horses weighing at least 200 kg of either sex admitted to our veterinary teaching hospital for elective surgery were considered for inclusion. Prior to inclusion, the history of the horse was taken, and a clinical examination was performed. Horses assigned an American Society of Anesthesiologist (ASA) physical status grade I–II were included after informing owner or trainer consent. Exclusion criteria were if sedation was deemed inadequate for safe induction of anesthesia after a total dose of 0.12 mg kg^−1^ romifidine (Sedivet vet, Boehringer Ingelheim, Vetmedica GmbH Ingelheim/Rhein, Germany) IV, if the placement of arterial line was unsuccessful, and if MAP was below 60 mm Hg at the start of the study. If MAP fell below 50 mm Hg at any time during the study infusion of dobutamine 0.5 μg kg^−1^min^−1^ was started, further data were excluded.

### Preparation, anesthesia, and instrumentation

Animals admitted for elective surgery were hospitalized overnight, with withdrawal of food overnight (9–13 h) but with free access to water. All horses had a physical examination the day before or at the day of anesthesia. Horses were weighed and an intravenous cannula (Secalon T, Merit Medical Ltd, Galway, Ireland) was placed into a jugular vein.

Horses were pre-medicated in the induction box with romifidine; start dose was based on the demeanor of the horse and thereafter given to effect at 0.02 mg kg^−1^ increments, with a maximum 0.12 mg kg^−1^ IV. The aim was adequate sedation for induction and the assessment of sedation was performed using subjective, clinical judgement. This would include lowering of the horse's head, dropped lower lip and unresponsiveness to movement and sound. Anesthesia was induced with ketamine (Ketamine Le Vet, Le Vet Beheer B.V, Holland) 2.5 mg kg^−1^ IV and midazolam (Midazolam, B. Braun Melsungen AG, Melsungen, Germany) 0.05 mg kg^−1^ IV. After induction of anesthesia and endotracheal intubation, the horse was placed on a padded surgical table in either lateral or dorsal recumbency and connected to the anesthesia machine (Tafoinus large animal ventilator, Ventronic Service Ltd). Anesthesia was maintained with isoflurane (Isoflo Vet, Zoetis, Florham, New Jersey, USA) in oxygen and medical air. The fresh gas flow (FGF) was set to 10 ml kg^−1^min^−1^ with FiO_2_ 40–60 % and the isoflurane vaporizer on 3.5 %. A multiparameter anesthetic monitor (Carescape B650, GE Healthcare Finland Oy, Helsinki Finland) was connected. Intermittent positive pressure ventilation (IPPV) was instituted with a respiratory rate of 10 min^−1^, I:E ratio of 1:2, and tidal volume was adjusted to achieve a PE'CO_2_ of 5.0–7.5 kPa. A 20–22 G catheter (BD Venflon Pro safety, Becton Dickinson Infusion Therapy, Helsingborg, Sweden) was placed in the facial artery. Before initiating the measurement of invasive arterial blood pressure, the pressure transducer (TrueWave, Edwards Lifesciences LLC, One Edwards Way, Irvine, CA, USA) was placed in level with the heart and zeroed to atmospheric pressure. Throughout the anesthesia, all horses were provided an infusion of Ringers Lactate (Infusolec, Decra Veterinary products AS, Oslo, Norway) 5–10 ml kg^−1^h^−1^ intravenously. An infusion of dexmedetomidine (Dexdomitor, Orion Pharma, Oslo, Norway) 1 μg kg^−1^h^−1^ given by a syringe driver (Agilia SP MC WIFI NO, Fresenius Kabi AG, Bad Homburg, Germany) was started as the endotracheal tube was connected to the anesthetic machine.

### Clinical trial

**Figure 1:**
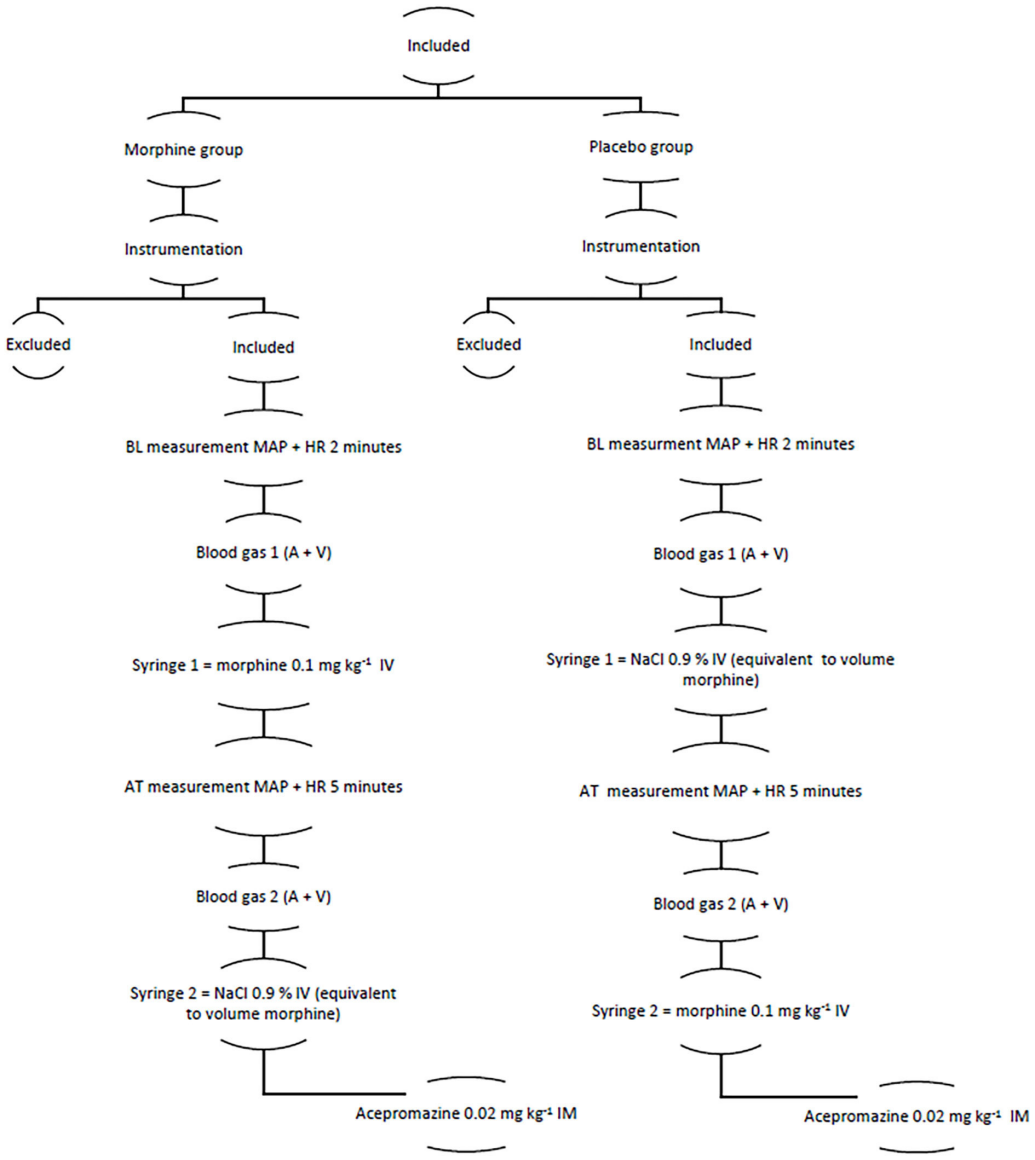
The figure showing the structure of the clinical trial A, arterial; AT, after treatment; BL, baseline; HR, heart rate; MAP, mean arterial pressure; V, venous.

An outline of the study is presented in Figure 1.

After an instrumentation period of 15–20 min, HR and MAP were sampled during a two-minute period as a baseline, and venous blood was sampled from the right or left jugular vein and arterial blood from the intra-arterial catheter. Thereafter, either morphine (Morfin Takeda, Norway Holding AS, Asker, Norway) of 0.1 mg kg^−1^ IV or an equivalent volume of NaCl 0.9% IV (Natriumklorid, B. Braun Melsungen AG, Melsungen, Germany) was administered as a rapid bolus. Syringes were prepared and labeled based on prior randomization by a person not involved in the study. HR and MAP were then further sampled for 5 min, and venous blood was sampled from the right or left jugular vein and arterial blood from the intra-arterial catheter. The values were collected, both continuously with a computer software for clinical data collection (iCollect Version 5.0, GE Healthcare, Finland) and manually every minute. The venous and arterial samples were analyzed on a benchtop analyzer (Radiometer ABL 90 FLEX PLUS, Radiometer Medical, Copenhagen, Denmark) within 30 min from collection. After completion of data sampling, horses previously administered morphine were administered saline and vice versa.

The clinical trial was carried out by the same person (EH). Both the person responsible for the trial and the anesthetist responsible for case management were blinded to treatment. After administration of test drugs was finished, acepromazine (Plegicil Vet, Pharmaxim AB, Helsingborg, Sweden) of 0.02 mg kg^−1^ IM was administered as a part of the hospital standard anesthetic protocol. Thereafter, the trial was ended. The intended procedure was performed, and anesthesia and recovery were now up to the anesthetist attending the case.

OE (mL/dL) was calculated as follows:


$$\begin{array}{l} OE=\left(\left(1.34\;\times Hb\;\times \;SaO_{2\;}\right)+\left(0.023\;\times \;PaO_{2\;}\right)\right)\\ \;-\;\left(\left(1.34\;\times Hb\;\times \;SvO_{2\;}\right)+\left(0.023\;\times \;PvO_{2\;}\right)\right) \end{array}$$

Values of hemoglobin (Hb), SaO_2_, SvO_2_, PaO_2_, and PvO_2_ were obtained from arterial and venous blood. The median value of the four Hb values for every horse was used in the calculation.

The end tidal isoflurane concentration (EtISO) was manually noted every 10 min, and linear interpolation was used to find the EtISO at baseline and after the last measurement of IBP.

### Statistics

A graphical plot was made of the data and visually inspected. For data where a normal distribution was assumed, Student's t statistics were used and data presented as mean ± SD. For data where no specific distribution was assumed, Wilcoxon-signed rank test was used and data presented as a median and range.

For MAP, the difference between the minimum value during baseline and the minimum value for the observation period was calculated and termed delta MAP. Delta MAP was normalized to the minimum baseline value and is reported as a percentage. For HR, the difference between the mean HR during baseline and the mean HR for the observation period was calculated and termed delta HR. For OE, the difference between baseline OE and OE during the observation period was calculated and termed delta OE. Alpha was set to 0.05.

Confidence intervals 95% (CI) were calculated for the delta MAP, delta HR, and delta OE within groups, and for the difference between groups. Statistical analysis was performed using a statistical software JMP v.14.1.0 (SAS Institute Inc., Cary, NC, USA).

## Results

An owner consent was received in 38 horses. All horses recovered uneventfully and were discharged from the hospital. Two horses were not included in the study due to the lack of adequate sedation for induction after romifidine 0.12 mg kg^−1^ IV and a total of three horses were excluded, two due to hypotension and one because of a non-functional arterial catheter within 20 min from the start of the study. Two horses received thiopental (Pentacur, Abcur, Helsingborg, Sweden) of 1 mg kg^−1^ IV due to movement before the study was started and one horse received acepromazine of 0.02 mg kg^−1^ IM > 2 h prior to premedication; none of these three horses were excluded from the study. No horse received dobutamine due to MAP < 50 mmHg. In total, 33 horses completed the study, 17 in the morphine group, and 16 in the placebo group. Breeds included in the study are represented in Table 1. Demographic data and dose of anesthetic drugs are presented in Table 2.

**Table 1:** The table showing breed distribution for all horses and within each group.

**Breed**	**All horses**	**Morphine group**	**Placebo group**
Warm blooded riding horse	12	5	7
Norwegian cold-blooded trotter	5	3	2
Standardbred	4	1	3
Thoroughbred	3	2	1
Dole horse	2	1	1
Fjord horse	2	2	
Icelandic horse	2	1	1
Friesian horse	1	1	
Northlands horse	1		1
Quarter horse	1	1	

**Table 2:** The table showing demographic data and anesthetic drug doses for all horses and within each group.

		**All horses**	**Morphine group**	**Placebo group**
Age (mean ± SD)		6.4 ± 4.2 years	5.3 ±3.6 years	6.8 ± 4.5 years
BW (mean ± SD)		501 ± 82 kg	484 ± 66 kg	514 ± 94 kg
Sex	Mare	14	7	7
	Stallion	12	6	6
	Gelding	7	4	3
				
Total dose romifidine (mean ± SD)		0.09 ± 0.02 mg kg^−1^	0.09 ± 0.02 mg kg^−1^	0.09 ± 0.02 mg kg^−1^
End-tidal isoflurane concentration (median, range) baseline		1.1 (0.8–1.3) %	1.1 (1.0–1.3) %	1.1 (0.8–1.3) %
End-tidal isoflurane concentration (median, range) after final IBP measurement		1.2 (1.1–1.4) %.	1.2 (1.1–1.4) %	1.2 (1.2–1.4) %

IBP, invasive blood pressure; SD, standards deviation.

Due to software (iCollect) failure, only 20 horses had continuous data collection, nine in the morphine group, and eleven in the placebo group. For the remaining horses, manually recorded data were used in the analysis.

**Figure 2:**
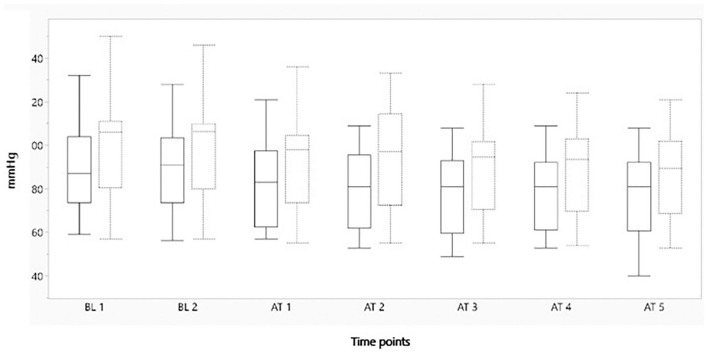
Boxplot showing minimum mean arterial blood pressure (mmHg) on the y-axis, before and after administration of either morphine or NaCl IV. The x-axis represents two baseline measurements with 1 min interval and five measurements after administration with 1 min interval. The solid line represents the morphine group and the staple line the placebo group. Boxplot characteristics: upper line = maximum value, excluding outliers, upper line of box = Q3 (75th percentile); middle line in box = median: lower line in box = Q1 (25th percentile) lower line = minimum value excluding outliers. AT, after treatment; BT, baseline. 1–5 = minutes before or after treatment.

**Figure 3:**
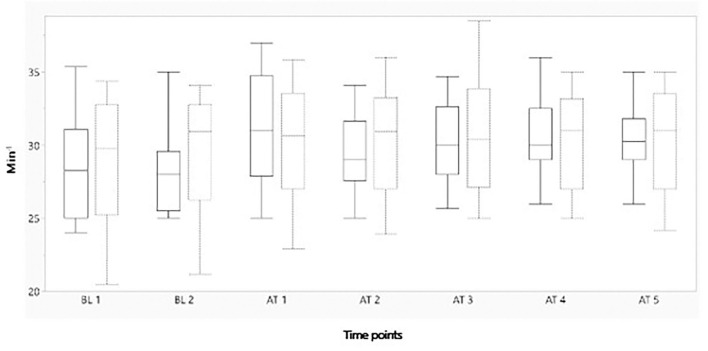
Boxplot showing mean heart rate (min^−1^) on the y-axis, before and after administration of either morphine or NaCl IV. The x-axis represents two baseline measurements with 1 min interval and five measurements after administration with 1 min interval. The solid line represents the morphine group and the staple line the placebo group. Boxplot characteristics: upper line = maximum value, excluding outliers, upper line of box = Q3 (75th percentile); middle line in box = median: lower line in box = Q1 (25th percentile) lower line = minimum value excluding outliers. BT, baseline; AT, after treatment. 1–5= minutes before or after treatment.

**Figure 4:**
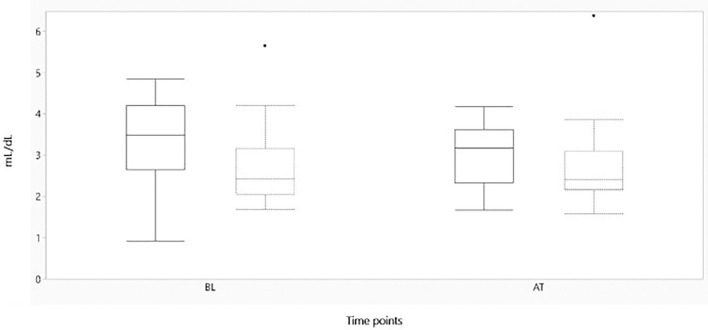
Boxplot showing oxygen extraction (mL/dL) on the y-axis, before and after administration of either morphine or NaCl IV. The x-axis represents baseline and after treatment of either morphine or NaCl 0.9% IV. The solid line represents the morphine group and the staple line the placebo group. Boxplot characteristics: upper line = maximum value, excluding outliers, upper line of box = Q3 (75th percentile); middle line in box = median: lower line in box = Q1 (25th percentile) lower line = minimum value excluding outliers, dots = outliers. AT, after treatment; BL, baseline.

Figures 2–4 represent, respectively, MAP, HR, and OE for the baseline and observation period. MAP and HR were higher in the placebo group compared to the morphine group throughout the study.

The 95% confidence intervals for delta MAP, delta HR, and delta OE and the confidence interval for the difference between the two groups for these variables are presented in Table 3. No significant differences between morphine and placebo groups were found for any of the parameters (Table 3).

**Table 3:** The table shows the 95 % confidence interval for the change in minimum mean arterial pressure, mean heart rate and oxygen extraction before and after administration of either morphine or NaCl IV.

	**Group**	**95 % CI within group**	**95 % CI (*p*-value) between groups**
Min MAP delta (%)	M	−20.5 to −9.0	−5.5 to 7.6 (0.96)
	P	−17.4 to −10.1	
Mean HR delta (min^−1^)	M	0.6–3.1	−2.3 to 0.7 (0.38)
	P	0.2–2.0	
OE delta (mL/dL)	M	−0.1 to 0.6	−0.7 to 0.2 (0.29)
	P	−0.2 to 0.3	
			

The p-values and the confidence intervals for the difference between the two groups is also shown. BL, baseline; CI, confidence interval; HR, heart rate; M, morphine; MAP, mean arterial pressure; OE, oxygen extraction; P, placebo.

The minimum MAP of a 6 years old, Norwegian cold-blooded trotter gelding in the morphine group had a minimum MAP of 76 mm Hg before administration and minimum MAP of 40 mm Hg during the observation period. The OE and HR for the same horse were almost unchanged between baseline and the observation period.

## Discussion

The cardiovascular effect of morphine after intravenous injection in horses is only investigated to some extent (2, 6–11), and this is one of the first studies investigating the cardiovascular effect of morphine in horses during general anesthesia. This study established 95% confidence intervals for the possible effect of morphine upon MAP, HR, and OE in anesthetized horses. We consider the confidence intervals to be quite narrow, and all values included in the confidence intervals are likely of little clinical significance. This thus supports the conclusion that the potential cardiovascular consequence of administration of 0.1 mg kg^−1^ morphine IV to horses during general anesthesia in general seems negligible. This is also a likely explanation that why no significant cardiovascular effects were found in previous studies (2, 8, 9).

When testing for statistical significance or establishing a confidence interval, it is usually the central tendency of the general populations that is tested (17). In the clinical setting, individuals are anesthetized, while the central tendency in a population is compared statistically. Interestingly, in this study, one horse in the morphine group had a decrease in minimum MAP around 50%. This can very well be a coincidental finding, or an individual with an idiosyncratic reaction to morphine. In total, three Norwegian cold-blooded trotters were included in the morphine group and no other had the same decrease in MAP. However, a much larger sample size would be needed to estimate the proportion of horses responding like this to morphine. This highlights the difficulty in revealing rare clinical events of importance, but it emphasizes the general wisdom of performing intravenous injection under anesthesia slowly and observing the response.

All values encompassed in the 95% confidence interval for delta mean MAP in both groups imply a clinically relevant change in MAP in the start of the anesthesia, but not so for HR or OE. A likely cause is the cardiovascular changes caused by a rising blood isoflurane concentrations. The quite narrow confidence interval of delta OE indicate that the global DO_2_ was adequate during this period.

Compared to other studies examining morphine's cardiovascular effects in horses, this study differs in multiple ways. Our study investigated morphine's cardiovascular effect immediately after administration whereby other studies investigated the effect 2 (8), 10 (2, 11), and 30 (9) min after administration. The study measuring the IBP and HR 2 min after administration of morphine or placebo only did one measurement within the first 5 min (8) whereby our study do one every minute the first 5 min. All horses in this study were premedicated, induced, and maintained with romifidine, ketamine, midazolam, isoflurane, and dexmedetomidine continuous rate infusion (CRI). The rationale behind the chosen drugs was to use drugs normally used for general anesthesia in horses at our hospital but also generally used for equine anesthesia. Similar drugs were used in other studies (2, 8, 9); however, only one previous study (18) used the combination of isoflurane and dexmedetomidine for the maintenance of anesthesia. The dose of morphine used differs between studies. One study used two different doses (8); however, both lower than used in this study. The other studies used same or higher doses than used in our study (2, 9, 11). One study in horses found a significant decrease in MAP after intravenous injection of morphine (11) and similar decreases have been shown in other species (3–5). In dogs and cats, an increased histamine release was documented concurrently with the cardiovascular changes (3–5). However, doses used in these studies were high, 1 mg kg^−1^, 2–4 mg kg^1^, 2–6 mg kg^−1^, and 0.2 mg kg^−1^, respectively, (3–5, 11) compared to this study. A progressively increase of the cardiovascular effect with increasing morphine doses can, therefore, not be excluded when comparing to existing studies in horses. The results in dogs and cats might indicate a dose-response effect of morphine on cardiovascular parameters, and that the conventional doses used in horses might be too low to elicit such effects. Two studies investigated the cardiovascular response in anesthetized horses after morphine 0.15 mg kg^−1^ bolus followed by and infusion of 0.1 mg kg^−1^ h^−1^ with varying results (18, 19). One study found a significant decrease in HR and increase in OE ratio, but no change in MAP compared to baseline (18). The other study found no significant change in HR or MAP between the morphine and placebo group and, the need of dobutamine infusion was similar between groups (19).

By coincidence, the baseline measurement of minimum MAP differed between the groups. Therefore, the change in minimum MAP between baseline and after treatment was normalized for both groups to ease for statistical comparison and interpretation.

In this study, both *p-*values and confidence intervals were used. A *p*-value can only answer the dichotomous question whether a significant predetermined alpha level is reached or not in a hypothesis testing situation. A confidence interval will capture the true central tendency of the population with a certainty specified as a percentage. Thus, there is more information in a confidence interval. The approach of using confidence intervals has increasingly been advocated (20, 21). The advantage is illustrated in the current study where quite narrow confidence intervals for the variables of interest were found giving the opportunity to state more clearly that whether the possible change is of clinical interest or not instead of only being able to state that no significant effect was found.

Prior to the study, we expected a hypotensive effect upon MAP. The minimum value of MAP within the baseline and observation period was used for comparison to ensure that a possible rapidly decreasing MAP, which then stabilized again, still would be taken fully into account. If a mean had been used, the effect of nadir MAP would be less since the value would be to some extent canceled by the other values. If HR or OE was influenced by morphine, a change in both directions was considered likely, and thus the mean value was used.

Horses in either lateral or dorsal recumbency were included in this study since it reflects the clinical nature. Both recumbencies affect the blood pressure (22) and cardiac index CI (23) during halothane anesthesia. The greatest effect on the blood pressure is reported in dorsal recumbency; however, no statistically significant difference between the positions was found (22). Also, a non-significant decrease in CI is reported when changing position from lateral to dorsal recumbency (23). No horse in our study had a change in position during the study.

Several limitations for this study exist. Dobutamine administration to the horse with minimum MAP of 40 mmHg during the observation period was not initiated since it was revealed when the data were interpreted after the anesthesia. The dose of morphine may have been too low to elicit a cardiovascular effect. However, the dose used is commonly administered to horses in the perianesthetic period. Also, a delayed cardiovascular effect extending beyond the 5 min study period cannot be excluded. Only three different parameters were used to evaluate the cardiovascular change after administration of morphine IV. Other parameters could have been of interest when investigating the cardiovascular changes and tissue perfusion, for example Qt, perfusion index, or plethysmography variability. Also, measurement of histamine plasma concentration would have been of interest since an increase have been described in other species (3–5). However, due to the clinical nature of this study, only MAP, HR, and OE were deemed to be most suitable. The use of dexmedetomidine might have influenced the cardiovascular response since bradycardia, hypertension (15, 24), and increased OE (15) are reported in horses. However, all horses received the same dose of dexmedetomidine, and the possible effect should be similar between the groups.

## Conclusion

The effects from administration of morphine 0.1 mg kg^−1^ IV upon HR, MAP, and OE to horses anesthetized with volatile anesthetics and dexmedetomidine are small and without clinical relevance.

## Data availability statement

The raw data supporting the conclusions of this article will be made available by the authors, without undue reservation.

## Ethics statement

The animal study was reviewed and approved by the National Animal Research Authority, FOTS id 14/04723-66. Written informed consent was obtained from the owners for the participation of their animals in this study.

## Author contributions

EH planned and carried out the study with supervision of AL and HH. EH did the data collection, data-analysis, and writing of the manuscript with supervision of AL and HH. All authors have read and approved the final version of the manuscript.

## Funding

This study was funded by the Norwegian University of Life Sciences.

## Conflict of interest

The authors declare that the research was conducted in the absence of any commercial or financial relationships that could be construed as a potential conflict of interest.

## Publisher's note

All claims expressed in this article are solely those of the authors and do not necessarily represent those of their affiliated organizations, or those of the publisher, the editors and the reviewers. Any product that may be evaluated in this article, or claim that may be made by its manufacturer, is not guaranteed or endorsed by the publisher.

## References

[1] Riviere JE Papich MG Adams HR. Veterinary Pharmacology and Therapeutics, 9th Edn. Ames, IW: Wiley-Blackwell Press (2009).

[2] Love EJ Geoffrey Lane J Murison PJ. Morphine administration in horses anaesthetized for upper respiratory tract surgery. Vet Anesth Analg. (2006) 3:179–88. 10.1111/j.1467-2995.2005.00247.x 16634944

[3] Morris KJ Zeppa R. Histamine-induced hypotension due to morphine and arjonad in the dog. J Surg Res. (1963) 6:313–17. 10.1016/S0022-4804(63)80012-8 14051999

[4] Evans AGJ Nasmyth PA Stewart HC. The Fall of Blood Pressure Caused by Intravenous Morphine in the Rat and the Cat. Brit J Pharmacol. (1952) 7:542–52. 10.1111/j.1476-5381.1952.tb00720.x 13019021 PMC1509299

[5] Kayaalpand SO Kaymakcalan SA. Comparative study of the effects of morphine in unanaesthetized and anaesthetized cats. Brit J Pharmacol. (1966) 26:196–204. 10.1111/j.1476-5381.1966.tb01821.x 4162141 PMC1510761

[6] Kalpravidh M Lumb WV Wright M Heath RB. Effects of butorphanol, flunixin, levorphanol, morphine, and xylazine in ponies. Am J Vet Res. (1984) 2:217–23. 6711945

[7] Muir WWSR Sheehan WC. Cardiopulmonary effects of narcotic agonists and a partial agonist in horses. Am J Vet Res. (1978) 10:1632–5. 717878

[8] Nolan AM Chambers JP Hale GJ. The Cardiorespiratory effects of morphine and butorphanol in horses anaesthetised under clinical conditions. J Vet Anesth. (1991) 1:19–24. 10.1111/j.1467-2995.1991.tb00007.x

[9] Mircica E Clutton RE Kyles KW Blissitt KJ. Problems associated with perioperative morphine in horses: a retrospective case analysis. Vet Anaesth Analg. (2003) 3:147–55. 10.1046/j.1467-2995.2003.00092.x 14498846

[10] Hamamoto-Hardman BD Steffey EP Weiner D McKemie DS Kass P Knych HK. Pharmacokinetics and selected pharmacodynamics of morphine and its active metabolites in horses after intravenous administration of four doses. J Vet Pharmacol Ther. (2019) 4:401–10. 10.1111/jvp.12759 30919469

[11] Ruiz-Lopez P Morgaz J Quiros-Carmona S Navarrete-Calvo R Dominguez JM Gomez-Villamandos RJ. Parasympathetic tone changes in anesthetized horses after surgical stimulation, and morphine, ketamine, and dobutamine administration. Animals. (2022) 8:1–10. 10.3390/ani12081038 35454284 PMC9027407

[12] Schauvliege S Gasthuys F. Drugs for cardiovascular support in anesthetized horses. Vet Clin North Am Equine Pract. (2013) 1:19–49. 10.1016/j.cveq.2012.11.011 23498044

[13] Tranquilli WJ Thurmon JC Grimm KA. Lumb and Jones' Veterinary Anesthesia and Analgesia, 4th Edn. Ames, IW: Blackwell Press (2007).

[14] Johnston GM Eastment JK Wood J Taylor PM. The confidential enquiry into perioperative equine fatalities (CEPEF): mortality results of phases 1 and 2. Vet Anaesth Analg. (2002) 4:159–70. 10.1046/j.1467-2995.2002.00106.x 28404360

[15] Risberg AI Ranheim B Krontveit RI Lervik A Haga HA. The cardiovascular status of isoflurane-anaesthetized horses with and without dexmedetomidine constant rate infusion evaluated at equivalent depths of anesthesia. Vet Anaesth Analg. (2016) 4:412–23. 10.1111/vaa.12315 26488527

[16] Silance PG Simon C Vincent JL. The relation between cardiac index and oxygen extraction in acutely III patients. Chest J. (1994) 4:1190–97. 10.1378/chest.105.4.1190 8162748

[17] Mishra P Pandey CM Singh U Gupta A Sahu C Keshri A. Descriptive statistics and normality test for statistical data. Ann Card Anesth. (2019) 1:67–72. 10.4103/aca.ACA_157_18 30648682 PMC6350423

[18] Benmansour P Husulak ML Bracamonte JL Beazley SG Withnall E Duke-Novakovski T. Cardiopulmonary effects of an infusion of remifentanil or morphine in horses anesthetized with isoflurane and dexmedetomidine. Vet Anaesth Analg. (2014) 4:346–56. 10.1111/vaa.12149 24673858

[19] Clark L Clutton RE Blissitt KJ Chase-Topping ME. Effects of peri-operative morphine administration during halothane anaesthesia in horses. Vet Anaesth Analg. (2005) 1:10–5. 10.1111/j.1467-2995.2004.00174.x 15663734

[20] du Prel JB Hommel G Rohrig B Blettner M. Confidence interval or p-value? Part 4 of a series on evaluation of scientific publications. Dtsch Arztebl Int. (2009) 19:335–9. 10.3238/arztebl.2009.0335 19547734 PMC2689604

[21] Amrhein V Greenland S McShane B. Scientists rise up against statistical significance. Nature. (2019) 7748:305–7. 10.1038/d41586-019-00857-9 30894741

[22] Gasthuys F De Moor A Parmentier D. Haemodynamic effects of change in position and respiratory mode during a standard halothane anaesthesia in ponies. J Vet Med Series A. (1991) 10:203–11. 10.1111/j.1439-0442.1991.tb01003.x 1907066

[23] Stegmann GF Littlejohn A. the effect of lateral and dorsal recumbency in cardiopulmonary function in the anaesthetized horse. J South Afr Vet Assoc. (1987) 1:21–7. 3112395

[24] Marcilla MG Schauvliege S Segaert S Duchateau L Gasthuys F. Influence of a constant rate infusion of dexmedetomidine on cardiopulmonary function and recovery quality in isoflurane anaesthetized horses. Vet Anaesth Analg. (2012) 1:49–58. 10.1111/j.1467-2995.2011.00672.x 22151875

